# Antegrade axillary arterial perfusion in 3D endoscopic minimally-invasive mitral valve surgery

**DOI:** 10.3389/fcvm.2022.980074

**Published:** 2022-09-30

**Authors:** Johannes Petersen, Shiho Naito, Benjamin Kloth, Simon Pecha, Svante Zipfel, Yousuf Alassar, Christian Detter, Lenard Conradi, Hermann Reichenspurner, Evaldas Girdauskas

**Affiliations:** ^1^Department of Cardiovascular Surgery, University Heart & Vascular Center, UKE Hamburg, Hamburg, Germany; ^2^Department of Cardiothoracic Surgery, Augsburg University Hospital, Augsburg, Germany

**Keywords:** minimally-invasive surgery, mitral valve, antegrade perfusion, axillaris cannulation, mitral vale surgery

## Abstract

**Background:**

Minimally-invasive (MIS) mitral valve (MV) surgery has become standard therapy in many cardiac surgery centers. While femoral arterial perfusion is the preferred cannulation strategy in MIS mitral valve surgery, retrograde arterial perfusion is known to be associated with an increased risk for cerebral atheroembolism, particularly in atherosclerosis patients. Therefore, antegrade perfusion may be beneficial in such cases. This analysis aimed to compare outcomes of antegrade axillary vs. retrograde femoral perfusion in the MIS mitral valve surgery.

**Methods:**

This analysis includes 50 consecutive patients who underwent MIS between 2016 and 2020 using arterial cannulation of right axillary artery (Group A) due to severe aortic arteriosclerosis. Perioperative outcomes of the study group were compared with a historical control group of retrograde femoral perfusion (Group F) which was adjusted for age and gender (*n* = 50). Primary endpoint of the study was in-hospital mortality and perioperative cerebrovascular events.

**Results:**

Patients in group A had a significantly higher perioperative risk as compared to Group F (EuroSCORE II: 3.9 ± 2.5 vs. 1.6 ± 1.5; *p* = 0.001; STS-Score: 2.1 ± 1.4 vs. 1.3 ± 0.6; *p* = 0.023). Cardiopulmonary bypass time (group A: 172 ± 46; group F: 178 ± 51 min; *p* = 0.627) and duration of surgery (group A: 260 ± 65; group F: 257 ± 69 min; *p* = 0.870) were similar. However, aortic cross clamp time was significantly shorter in group A as compared to group F (86 ± 20 vs. 111 ± 29 min, *p* < 0.001). There was no perioperative stroke in either groups. In-hospital mortality was similar in both groups (group A: 1 patient; group F: 0 patients; *p* = 0.289). In group A, one patient required central aortic repair due to intraoperative aortic dissection. No further cardiovascular events occurred in Group A patients.

**Conclusion:**

Selective use of antegrade axillary artery perfusion in patients with systemic atherosclerosis shows similar in-hospital outcomes as compared to lower risk patients undergoing retrograde femoral perfusion. Patients with higher perioperative risk and severe atherosclerosis can be safely treated *via* the minimally invasive approach with antegrade axillary perfusion.

## Introduction

Mitral Valve (MV) surgery has become the therapy of choice in severe MV disease and has been shown to result in low perioperative mortality and excellent long-term results (1). The introduction of minimally invasive (MIS) access in MV surgery has decreased surgical trauma and has resulted in faster postoperative recovery as well as higher patient satisfaction (1–3). While femoral arterial perfusion is the preferred cannulation strategy in MIS retrograde arterial perfusion is known to be associated with an increased complication risk in patients with atherosclerosis (4, 5). Therefore, antegrade perfusion may be beneficial in such situations. Different techniques have been proposed for antegrade perfusion during MIS. While Murzi et al. were able to show that antegrade direct cannulation of the aorta in MIC MV surgery resulted in a significant reduction of stroke and delirium (4, 6), a small cases series presented carotid artery cannulation for antegrade perfusion during MIC MV surgery (7). However, there are limited systematic reports regarding axillary artery cannulation for antegrade perfusion for MIS. This analysis aimed to compare postoperative outcomes of direct axillary artery perfusion in a consecutive cohort of patients with severe systemic atherosclerosis undergoing MIS and compare it to a control group of patients who had retrograde femoral perfusion.

## Patients and methods

All patient data were anonymized and analyzed retrospectively. Formal consent from the patients was not obtained due to anonymity of the database. This analysis assessed perioperative outcomes of 50 consecutive patients who underwent MIS between July 2016 and January 2020 using a direct arterial cannulation of the right axillary artery (Group A). Perioperative outcomes of the study group were compared with a historical (January 2011–April 2018) age- and gender-adjusted control group of patients who had a retrograde femoral perfusion during MIS mitral valve surgery (Group F) (*n* = 50). Primary study endpoint was in-hospital mortality. Secondary endpoint was the rate of perioperative cerebrovascular events in both study groups.

### Preoperative protocol for minimally-invasive surgery

**FIGURE 1:**
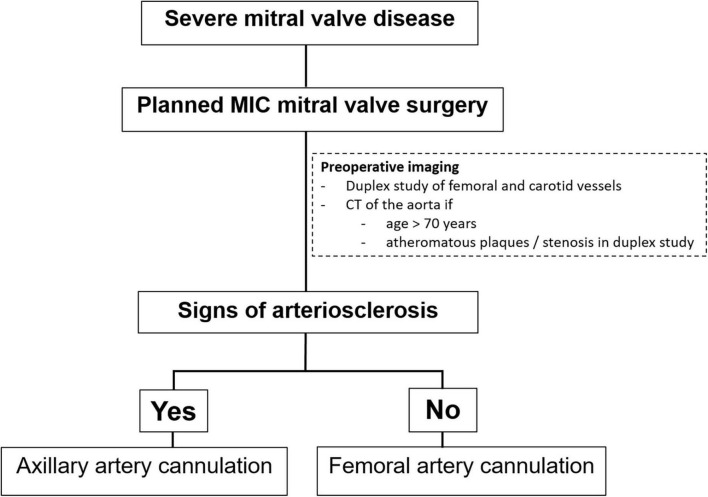
Treatment strategy at our institution in patients with severe mitral valve (MV) disease planned for a minimally-invasive (MIC) mitral valve surgery.

**FIGURE 2:**
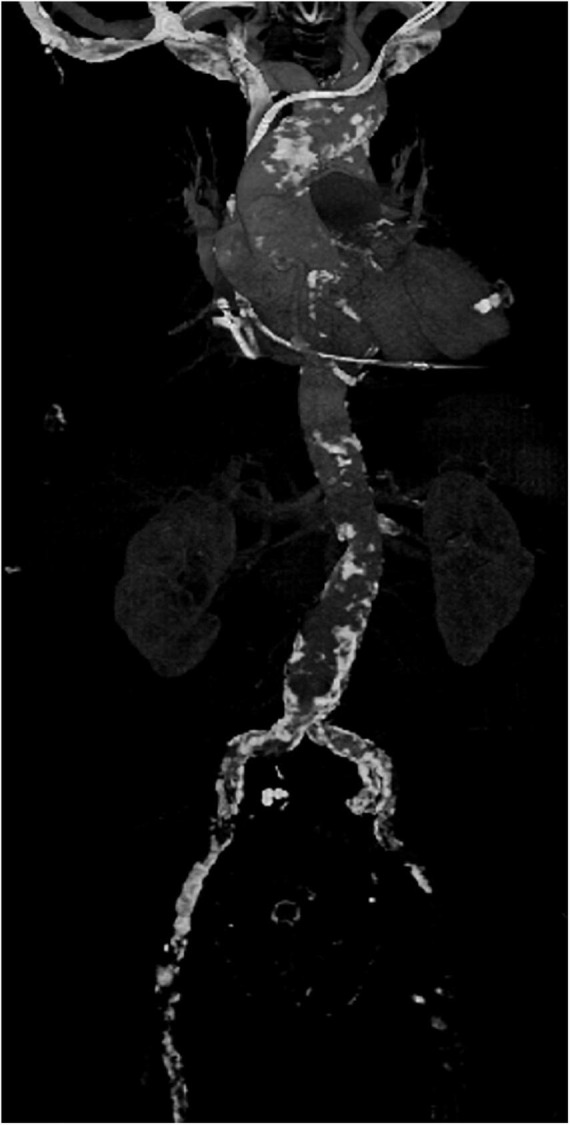
Example of a computed tomography (CT) scan of the aorta of a patient with severe arteriosclerosis in the axillary artery, aortic arch abdominal aorta and especially the femoral vessels in which minimally-invasive surgery was performed by using an antegrade arterial perfusion *via* arteria axillaris.

**FIGURE 3:**
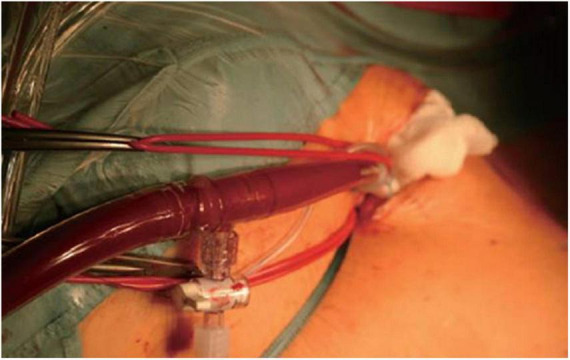
Intraoperative situs of antegrade arterial perfusion *via* axillaris artery in minimally-invasive mitral valve surgery.

In our institution, all patients presenting with severe MV disease (regurgitation or stenosis) are considered potential candidates for minimally-invasive access (Figure 1). If concomitant procedures necessitate median sternotomy access (e.g., coronary artery bypass graft, aortic valve replacement, replacement of the ascending aorta), the patient is scheduled for conventional sternotomy with a limited skin incision. In case of isolated mitral valve surgery (repair or replacement) as well as in need of simultaneous tricuspid valve repair, closure of left atrial appendage or surgical ablation, minimally invasive surgery is planned. In this setting we routinely use right anterolateral minithoracotomy with soft tissue retractor and 3D fully-endoscopic approach. In such patients (group A as well as group F), preoperative imaging includes a duplex evaluation of femoral and carotid vessels. If atheromatous plaques or a stenosis is present in the sonography, risk factors for arteriosclerosis are present or if the patient is ≥70 years, a native computed tomography (CT) scan of the thoracic and abdominal aorta is performed. With this systematic approach unnecessary radiation exposure in younger patients (<70 years) will be prevented. If there are no signs of arteriosclerosis a retrograde arterial perfusion is performed by direct cannulation of the arteria femoralis. In case of systemic arteriosclerosis in the thoracic or/and abdominal aorta in the CT scan (Figure 2), an antegrade arterial perfusion *via* axillary artery is performed (Figure 3).

### Procedural steps of axillary artery cannulation

The procedural steps of axillary artery cannulation are outlined in Supplementary Video 1. Following a 3 cm infraclavicular incision, the axillary artery is identified. After placement of cannlation sutures, direct puncture of the axillary artery is performed using Seldinger’s technique. Echocardiographic visualization of the wire is crucial to avoid vascular injury. After placing the dilatator over the wire, a 5–8 mm incision of the axillary artery is performed. Afterward, the arterial cannula is carefully placed 1.5–2 cm into the vessel avoiding any resistance. Depending of the size of the axillary artery, we use a 18 Fr. or 20 Fr. short-tip Medtronic cannula. After fixation of the cannula, another stay suture is placed at the skin level to prevent dislocation of the cannula during cardiopulmonary bypass (CPB) perfusion.

### Analysis of primary and secondary endpoints

Baseline and perioperative variables were collected retrospectively from our institutional electronic patient records and were entered into a standardized database. Intraoperative complications as well as in-hospital death were analyzed. Further postoperative complications such as neurovascular complications (e.g., stroke) or bleeding requiring redo surgery, pneumothorax, and access site complications (e.g., wound healing disorder, bleeding complications) were recorded.

### Statistical analysis

Categorical variables are expressed as frequencies and percentages throughout the manuscript and comparisons were made using chi-square test or Fisher’s exact test, as appropriate. Normally distributed continuous variables are presented as mean ± standard deviation. All reported *p*-values are two-sided and *p*-values of 0.05 or less were considered statistically significant. All statistical analyses were accomplished using Excel 16.21 (Microsoft, USA) and IBM SPSS 23 software (IBM Corp., New York, NY, USA).

## Results

### Preoperative characteristics

Preoperative characteristics of study and control groups are outlined in Table 1. Patients in both groups had similar age (i.e., group A: 74.2 ± 5.8 vs. group F: 73.9 ± 2.3; *p* = 0.829) and gender (58% males in both groups, *p* = 1.000). Patients in both groups presented with typical cardiovascular risk factors, while patients in group A had a significantly higher prevalence of diabetes (17% vs. 4.4%; *p* = 0.041) and were more frequently obese (26.3 ± 3.9 kg/m^2^ vs. 24.4 ± 3.6 kg/m^2^; *p* = 0.019). Four patients in group A suffered from infective endocarditis vs. one patient in the group F (*p* = 0.075). Furthermore, three patients in group A had status post stroke vs. one patient in group F (*p* = 0.306). Atrial fibrillation was present in 40% of the patients (group A: 54%; group F: 26%; *p* = 0.013), out of which 11 patients had paroxysmal or persistent atrial fibrillation. Left ventricular ejection fraction at baseline was significantly lower in group A vs. group F (49.0% ± 9.3% vs. 55.4% ± 8.1%, respectively, *p* = 0.001). Perioperative risk score values were significantly higher in group A vs. group F [EuroSCORE II: 3.9 ± 2.5 vs. 1.6 ± 1.5; *p* = 0.001; Society of Thoracic Surgeons (STS)-Score: 2.19 ± 1.49 vs. 1.31 ± 0.64; *p* = 0.023].

**TABLE 1:** Preoperative patient characteristics: Antegrade axillary perfusion (group A) vs. retrograde femoral perfusion (group F).

Patient characteristics	Group A (*n* = 50)	Group F (*n* = 50)	*p*-Value
Age (years)	74.2 ± 5.8	73.9 ± 2.3	0.829
Gender—male, *n* (%)	29 (58)	29 (58)	1.000
BMI, kg/m^2^	26.3 ± 3.9	24.4 ± 3.6	0.019
Arterial hypertension, *n* (%)	32 (64)	35 (70)	0.721
Diabetes, *n* (%)	7 (17)	2 (4.4)	0.041
Dyslipidemia, *n* (%)	12 (24)	12 (24)	1.000
Smoking, *n* (%)	6 (12)	2 (4)	0.144
Preoperative endocarditis, *n* (%)	4 (8)	1 (2)	0.075
Prior Stroke, n (%)	3 (6)	1 (2)	0.306
Atrial Fibrillation, *n* (%)	27 (54)	13 (26)	0.013
Baseline LVEF (%)	49.0 ± 9.3	55.4 ± 8.1	0.001
EuroSCORE II	3.99 ± 2.57	1.67 ± 1.58	0.001
STS-Score	2.19 ± 1.49	1.31 ± 0.64	0.023

BMI, Body Mass Index; EuroSCORE II, European System for Cardiac Operative Risk Evaluation; STS-Score, Society of Thoracic Surgeons-Score; LVEF, Left Ventricular Ejection Fraction.

### Intraoperative data

MV repair was performed in 86% patients in group A vs. 96% in group F (*p* = 0.067). Most common concomitant procedures were left atrial ablation (group A: 10% vs. group F: 12%; *p* = 1.000) and LAA closure with the AtriClip^®^ (Atricure Inc., West Chester, OH, USA) (group A: 8% vs. group F: 2%; *p* = 0.362) (Table 2). Two additional patients had closure of persistent foramen ovale in group F, while one patient in group A required simultaneous tricuspid valve repair. Cardiopulmonary bypass time (group A: 172 ± 46 min; group F: 178 ± 51 min; *p* = 0.627) and duration of surgery (group A: 260 ± 65 min; group F: 257 ± 69 min; *p* = 0.870) were similar in both groups. However, aortic cross clamp time was significantly shorter in group A as compared to group F (86 ± 20 min vs. 111 ± 29 min, *p* < 0.001). In group A, one patient required median sternotomy and central aortic repair due to intraoperative aortic dissection. This patient had a severe systemic atherosclerotic disease and most probably an intimal lesion was induced by the wire resulting in a type A aortic dissection. This event highlights that it is of highest importance to visualize the wire through transesophageal echocardiography and to push the wire forward without resistance. Despite this iatrogenic event the patient had an uneventful postoperative course and was discharged without neurological deficit 13 days following surgery. One patient in group A had an accidental dislocation of the axillary artery cannula during CPB and underwent emergent conversion to femoral cannulation. After this event, an additional stay suture of the cannula at the skin was placed for fixation. Consequently, no further dislocations of the cannula occurred. This particular patient had an uneventful postoperative course and was discharged home without neurological deficit 8 days following surgery.

**TABLE 2:** Intraoperative patient data.

Patient characteristics	Group A (*n* = 50)	Group F (*n* = 50)	*p*-Value
Mitral valve repair, *n* (%)	42 (86)	48 (96)	0.067
Concomitant procedures, *n* (%)			
Left atrial ablation, *n* (%)	5 (10)	6 (12)	1.000
LAA closure, *n* (%)	4 (8)	1 (2)	0.362
Closure of PFO, *n* (%)	0	2 (4)	0.494
Tricuspid valve repair, *n* (%)	1 (2)	0	1.000
Cardiopulmonary bypass time (min)	172 ± 46	178 ± 51	0.627
Aortic Cross-Clamp-time (min)	86 ± 20	111 ± 29	**<0.001**
Duration of surgery (min)	260 ± 65	257 ± 69	0.870

LAA, Left atrial appendage; PFO, Persistent Foramen Ovale. Significant values are highlighted in bold.

### Postoperative outcome

Duration of postoperative mechanical ventilation was significantly longer in group A vs. group F (10.1 ± 9.4 h vs. 5.9 ± 3.3 h; *p* = 0.045). However, total intensive care unit stay was similar in both groups (group A: 2.9 ± 2.6 days vs. group F: 2.1 ± 1.5 days; *p* = 0.113); Table 3. In-hospital stay was significantly longer in group A compared to group F (i.e., 10.0 ± 4.4 days vs. 7.1 ± 1.7 days, respectively, *p* < 0.001). In-hospital mortality was similar in both groups (group A: 1 patient; group F: 0 patients; *p* = 0.289). One patient in group A expired 5 days after surgery due to multi-organ failure. There was no in-hospital mortality in group F. In group A, a total of 6 patients required redo surgery due to bleeding vs. 4 patients in group F (*p* = 0.376). There were no perioperative stroke or vascular access-site complications in either group (Table 3). No further cardiovascular events occurred in the both groups.

**TABLE 3:** Postoperative patient data.

Patient characteristics	Group A (*n* = 50)	Group F (*n* = 50)	*p*-Value
Access site complications	0 (0%)	0 (0%)	1.000
Perioperative stroke	0 (0%)	0 (0%)	1.000
In-hospital mortality	1 (2%)	0 (0%)	0.289
Duration ICU stay (days)	2.9 ± 2.6	2.1 ± 1.5	0.113
Duration in-hospital stay (days)	10.0 ± 4.4	7.1 ± 1.7	<0.001

ICU, Intensive Care Unit.

## Discussion

This study highlights implications of systemic atherosclerosis on the arterial perfusion strategy during MIS. Antegrade axillary perfusion strategy enables minimally invasive MV surgery to be performed even in high-risk patients with higher perioperative risk scores and signs of systemic atherosclerosis.

Since the turn of the 20th century, MIS MV surgery has been of increasing interest in specialized cardiac centers (3). With the help of 3D fully-endoscopic visualization and robotic assistance minimally invasive technique developed toward a well-established surgical procedure (8–10). Establishment of cardiopulmonary bypass is usually achieved *via* femoral artery cannulation. However, there has been increasing concerns about retrograde arterial perfusion in older and high-surgical risk patients (4, 11–13). In 2010, a large analysis of MIS surgery from the STS-database showed an increased risk of perioperative strokes (13). Especially in patients with beating- or fibrillating-heart techniques or use of endoaortic balloons, neurologic events occurred more often. Furthermore, Grossi et al. showed that retrograde arterial perfusion resulted in significant more neurologic events (defined as permanent deficit, transient deficit greater than 24 h or a new lesion on cerebral imaging) compared to antegrade perfusion, especially in elderly patients (5). Another recent study confirmed an increased stroke rate in the setting of retrograde perfusion in high-risk reoperative mitral valve procedures (14). However, a meta-analysis and further single-center studies showed no differences in stroke rate between retrograde vs. antegrade arterial perfusion strategy (15–19).

Murzi et al. proposed an antegrade direct cannulation of the ascending aorta in MIS which led to significant reduction of stroke and delirium compared to a propensity-matched cohort with retrograde perfusion (4). However, this approach requires a more anterior and larger incision which reduces the benefits of the minimally invasive approach. Further, a case report suggested antegrade perfusion *via* the left axillary artery for combined endoartic balloon occlusion and perfusion during robotic mitral valve surgery (20). Bonaros et al. presented a report of two patients with severe arteriosclerosis of the abdominal aorta who underwent carotid artery cannulation for antegrade perfusion during MIS (7). Furthermore, Farivar et al. reported a case series of five patients in whom antegrade arterial perfusion *via* the right axillary artery was used during MIS (21). Most recently, Puiu et al. described a large cohort of 688 patients with cannulation of axillary artery (42% with direct cannulation) and concludes that cannulation of the right axillary through a vascular prosthetic graft reduces cannulation-related complications such as iatrogenic axillary artery dissection and stroke rates (22). However, the study population described is not comparable to our mitral valve cohort, since most of them had an aortic pathology which is at more risk of iatrogenic dissection. As opposite, in our MIS mitral valve cohort we routinely use the direct cannulation of the axillary artery in Seldinger technique supported by transesophageal echocardiography guidance which is of high importance in order to visualize the wire and check for a dissection membrane. Another pitfall of the direct axillary cannulation can be the dislocation of the arterial cannula which can be prevented by a stay suture placed at the skin level in order to stabilize the arterial cannula. This technique (Supplementary Video 1) has been shown to be fast and reproducible and can be used in patients with higher perioperative risk scores. Therefore, this procedure provides an excellent alternative in patients with higher-risk scores who nowadays are often referred for transcatheter mitral valve treatment (e.g., MitraClip, transapical transcatheter MV replacement) (23, 24).

Furthermore, assessment of the atherosclerotic burden is crucial when planning MIS. Preoperative CT screening is performed routinely at our site to detect arteriosclerosis of the aorta or ilio-femoral vasculature in all patients >70 years and in those with signs of generalized atherosclerotic disease (e.g., evidence of carotid or peripheral artery disease) in doppler studies. Preoperative CT screening prior to MIS has also been proposed by Moodley et al. (25). Their group performs CT scans in every patient planned for MV surgery, resulting in a change of surgical approach in 21% of patients. We believe that MIS can be safely be performed in the setting of severe systemic arteriosclerosis. However, antegrade arterial perfusion, e.g., using direct cannulation of right axillary artery, seems to be advisable in such cases.

### Limitations

This is a retrospective single center non-randomized analysis with all known limitations associated with such a study design and therefore drawing a final conclusion from this pilot study is limited. In addition, the patient sample is rather small. The main reason is that surgeons tend to treat patients with higher perioperative risk and severe atherosclerosis *via* median sternotomy or even refer those patients to transcatheter therapies. However, the focus of the study was to establish a standardized perfusion strategy in patients with a higher perioperative risk score undergoing MIS and to show its safety and reproducibility by analyzing perioperative outcomes. To validate this single center experience, a prospective multi-center study is necessary to confirm our current findings. Another limitation of the study is the comparison of the axillary group to a historical cohort with retrograde femoral perfusion. Nevertheless, group A includes patients with severe systemic atherosclerosis who are at higher surgical risk and therefore direct comparability is limited.

## Conclusion

In summary, the burden of arteriosclerosis is an important factor to consider before MIS MV surgery. Preoperative CT screening for aortic atherosclerosis seems to be reasonable in patients age >70 years and in those with signs of generalized atherosclerotic disease. Selective use of antegrade axillary artery perfusion in patients with systemic atherosclerosis shows similar in-hospital outcomes as compared to lower risk patients undergoing retrograde femoral perfusion. Patients with higher perioperative risk and severe atherosclerosis can be safely treated *via* the minimally invasive approach with antegrade axillary perfusion.

## Data availability statement

The raw data supporting the conclusions of this article will be made available by the authors, without undue reservation.

## Ethics statement

Ethical review and approval was not required for the study on human participants in accordance with the local legislation and institutional requirements. Written informed consent for participation was not required for this study in accordance with the national legislation and the institutional requirements.

## Author contributions

JP: conceptualization, data curation, formal analysis, methodology, investigation, and writing of original draft. SN: data curation, formal analysis, visualization, and review and editing of draft. BK and SP: methodology, resources, and review and editing of draft. SZ: resources and review and editing of draft. YA and CD: resources, supervision, and review and editing of draft. LC and HR: methodology, resources, supervision, and review and editing of draft. EG: project administration, conceptualization, supervision, and review and editing of draft. All authors contributed to the article and approved the submitted version.

## Supplementary material

Supplementary Video 1 Procedural steps of antegrade arterial perfusion *via* axillaris artery in minimally-invasive mitral valve surgery.
